# Inferring circadian gene regulatory relationships from gene expression data with a hybrid framework

**DOI:** 10.1186/s12859-023-05458-y

**Published:** 2023-09-26

**Authors:** Shuwen Hu, Yi Jing, Tao Li, You-Gan Wang, Zhenyu Liu, Jing Gao, Yu-Chu Tian

**Affiliations:** 1 School of Computer Science, Queensland University of Technology, Brisbane, QLD 4001 Australia; 2 Agriculture and Food, CSIRO, St Lucia, QLD 4067 Australia; 3 Faculty of Science, The University of New South Wales, Sydney, 2052 Australia; 4 School of Life Sciences, Inner Mongolia Agricultural University, Hohhot, 010018 China; 5 Institute for Learning Sciences and Teacher Education, Australian Catholic University, Brisbane, QLD 4000 Australia; 6 School of Computer and Information Engineering, Inner Mongolia Agriculture University, Hohhot, 010018 China

**Keywords:** Circadian gene, Gene regulatory relationships, Gene expression data, Fuzzy c-means clustering, Dynamic time warping

## Abstract

**Background:**

The central biological clock governs numerous facets of mammalian physiology, including sleep, metabolism, and immune system regulation. Understanding gene regulatory relationships is crucial for unravelling the mechanisms that underlie various cellular biological processes. While it is possible to infer circadian gene regulatory relationships from time-series gene expression data, relying solely on correlation-based inference may not provide sufficient information about causation. Moreover, gene expression data often have high dimensions but a limited number of observations, posing challenges in their analysis.

**Methods:**

In this paper, we introduce a new hybrid framework, referred to as Circadian Gene Regulatory Framework (CGRF), to infer circadian gene regulatory relationships from gene expression data of rats. The framework addresses the challenges of high-dimensional data by combining the fuzzy C-means clustering algorithm with dynamic time warping distance. Through this approach, we efficiently identify the clusters of genes related to the target gene. To determine the significance of genes within a specific cluster, we employ the Wilcoxon signed-rank test. Subsequently, we use a dynamic vector autoregressive method to analyze the selected significant gene expression profiles and reveal directed causal regulatory relationships based on partial correlation.

**Conclusion:**

The proposed CGRF framework offers a comprehensive and efficient solution for understanding circadian gene regulation. Circadian gene regulatory relationships are inferred from the gene expression data of rats based on the *Aanat* target gene. The results show that genes *Pde10a, Atp7b, Prok2, Per1, Rhobtb3* and *Dclk1* stand out, which have been known to be essential for the regulation of circadian activity. The potential relationships between genes *Tspan15, Eprs, Eml5* and *Fsbp* with a circadian rhythm need further experimental research.

**Supplementary Information:**

The online version of this paper contains supplementary materials available at 10.1186/s12859-023-05458-y.

## Introduction

Circadian rhythms are endogenous 24-h oscillations of behavioural and biological processes found in all kingdoms of life. Li et al. [1] have studied the relationship between circadian gene expression patterns in the human brain and disruption in major depressive disorder (MDD). They have showed that cyclic expression patterns are much weaker in the brains of patients with MDD. Zhang et al. [2] have highlighted critical, systemic, and surprising roles of the mammalian circadian clock and provided a blueprint for advancement in chronotherapy. In addition, Caba et al. [3] have shown the importance of circadian rhythms and clock genes in reproduction. Overall, inferring circadian gene regulatory relationships from time-series gene expression data is a cutting-edge research topic because gene regulatory relationships can be used to understand the molecular mechanism and disclose the essential rules of biological processed and reactions in organisms.

The aim of constructing correlation networks is to present relationships between genes [4]. After obtaining the correlation matrix from the gene expression data, a statistical test is required to test the significance of the correlation and thus decide whether possible edges are included in the gene association network. False discovery rate (FDR), which is defined as the expected proportion of false positives among the proposed edges, measures the significance of the relationships between genes. Therefore, it can be used to determine significant edges [5, 6]. A local FDR is an empirical Bayes estimator of the FDR. It is used to compute the posterior probability for an edge to be present or absent by taking account of the multiplicity in the simultaneous testing of edges [7]. However, the final network obtained by visualizing all significant edges is an undirected graph. Opgen-Rhein and Strimmer [8] have converted a correlation network into a partial correlation graph, thus identifying a directed acycli causal network as a subgraph of the partial correlation network. Furthermore, the gene expression data are usually multivariate time-series data. Martin et al. [9] have developed a method for inferring networks for time series microarray data to consider all possible networks matching a given time series dataset. Unlike traditional Bayesian networks that only consider steady-state data, Dynamic Bayesian networks (DBNs) use time information of genes more effectively [10–12]. Peng et al. [13] have proposed an approach called Sparse Partial Correlation Estimation (*space*) to select nonzero partial correlations. They claim that the *space* method performs better than the penalized likelihood method graphical LASSO [14] in both nonzero partial correlation selection and identification of nodes.

A major challenge in analyzing gene expression data is the low-sampling but high-dimensional data. The number of sampled observations is typically small compared to the number of considered genes. Some regularized methods are applied in the inference of large-scale gene association networks, e.g., ridge methods, partial least square, and least absolute shrinkage and selection operator (LASSO) [15]. Krämer et al. [16] have investigated a general framework that combines regularized regression methods (ridge and adaptive LASSO) with the estimation of graphical Gaussian models (GGMs) of high-dimensional microarray data. D’Angelo et al. [17] use principal-component analysis (PCA) and LASSO to identify gene interactions from the gene expression data. In addition, Barigozzi and Brownlees [18] have introduced network estimation for time series (NETS) under the assumption that vector auto-regressive (VAR) is spare. Furthermore, Ajmal and Madden [19] have shown that the LASSO method yields higher structural accuracy for graphs than G1DBM, which is proposed by [20]. These regularized methods are all applied to the construction of regulatory networks. Our work in this paper deals with the high-dimensional problem in a slightly different way. We construct a selective process before constructing gene regulatory relationships, implying the hybridisation of clustering algorithms and Dynamic Time Warping (DTW) distance.

Clustering is a common approach to select genes with similar expression fluctuation [21]. The clustering algorithm usually plays a role in data preprocessing before establishing the gene regulatory relationships [22]. The fuzzy C-means method is one of the most widely used clustering algorithms for microarrays [21]. From the clustering results, a specific cluster can be selected and then focused on, giving a feasible way for dimension reduction. However, from a selected cluster, some relationships would be omitted to establish specific gene regulatory relationships. This motivates our development of a hybrid framework in this work.

In this paper, we develop a new hybrid framework to infer circadian gene regulatory relationships from time-course microarray gene expression data. Microarray experiments result in 32,883 genes and 480 observations for each gene. We handle the high-dimensional problem by using the hybrid framework and establish the circadian gene regulatory relationships.

## Materials and methods

### Dataset collection

The data of time-series expression profiles of rats were obtained from the laboratory of Inner Mongolia Agricultural University (Inner Mongolia Autonomous Region Key Laboratory of Big Data Research and Application for Agricultural and Animal Husbandry) and uploaded to the NCBI SRA database [23]. The purpose of acquiring the data was to obtain the circadian genes that regulate the sleep of rats through the analysis of time-series gene expression data. A total number of 480 male rats (8-week-old) with an average body mass index of 180 g were selected from a rat farm in Qingdao, Shandong Province, China. All experimental rats were housed in a 100-square-meter independent room for two weeks. One pineal gland of the rat was sampled every three minutes during a complete circadian cycle from 7:00 am on November 15, 2020, to 7:00 am on November 16, 2020. This process continued for 24 h till the end of the experiment. The Institutional Experimental Animal Welfare and Ethics Committee of Inner Mongolia Agricultural University approved all procedures involving rats in this work. There were seven main steps for the experiment: (1) Take the rat to the test bench; (2) Euthanize the rat by decapitation; (3) Open the skull and take out the brain tissues; (4) Isolate the rhythm centre-pineal gland; (5) Identify second microstructure; (6) Remove rat pineal gland and put it in a 2 ml Corning Freezer Tube; (7) Label the sample and cryopreserved in liquid nitrogen immediately. More experimental details can be found in [23, 24].

Therefore, we sampled a total number of 480 pineal glands. Total RNA was extracted using the Biomed RNApure Rapid RNA Kit (RA103-02), and the RNA extraction results were detected by an Agilent Bioanalyzer 2100. Sequencing libraries were generated using the NEBNext® UltraTM RNA Library Prep Kit (NEB, USA). An index code was added to the attribute sequence for each sample. Library quality was assessed on an Agilent Bioanalyzer 2100 system. Index-encoded samples were clustered on the cBot cluster generation system using the TruSeq PE Cluster Kit v3 cBot HS. After cluster generation, the library was sequenced on the BGI DNBSEQ-T7RS platform, and 125 bp/150 bp paired-end reads were generated. Finally, dynamic time-series expression profiles of rats were obtained, which were composed of 32,883 genes, each with 480 observations.

### Framework

**Fig. 1:**
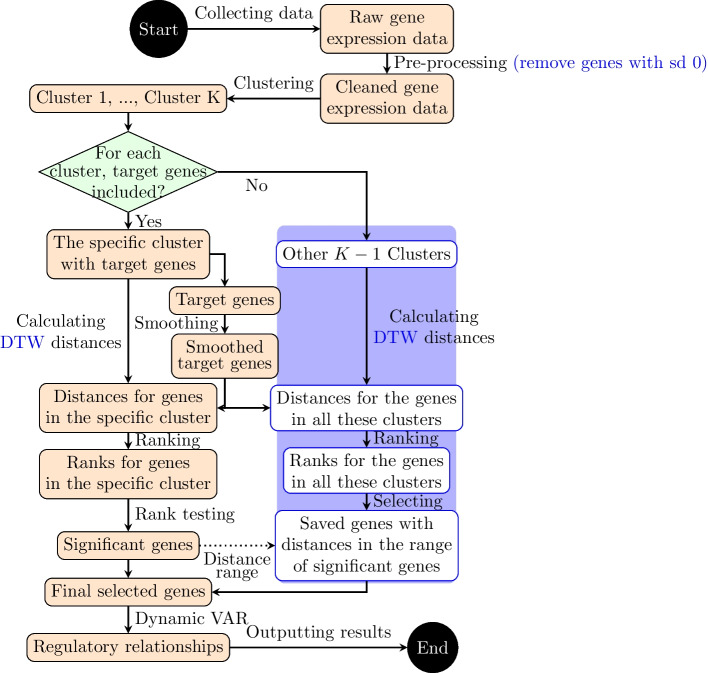
Logical block diagram of the new hybrid framework

A logical block diagram of our hybrid framework is depicted in Fig. 1. The main steps of using this framework are: (1) Use the fuzzy C-means clustering algorithm to cluster the gene expression data and select the specific cluster that includes target genes from the clustering results. (2) Identify and smooth the target genes that deserve further investigation. (3) Calculate the distances between all genes and the smoothed target genes. (4) Decide significant genes from the specific cluster based on the p-values of the Wilcoxon signed-rank test. (5) Save the genes that initially belong to other clusters with distances in the range of significant genes. (6) Use Dynamic vector autoregressive (VAR) models to construct regulatory relationships for the final selected gene expression profiles. The main novelty of this hybrid framework lies in its incorporation of clustering and distance analysis to examine time-series gene expression data, allowing for efficient clustering computation of the high-dimensional problem. Another novel aspect of the framework is the use of smoothing to capture the pattern of the target gene. Thus, the distances of all genes and the smoothness of the target genes are calculated to measure the similarity. A further advantage of the framework is that some genes that belong to other clusters but have distances in the range of significant genes are also considered. Therefore, no significant genes will be missed out.

### Fuzzy C-means clustering

Clustering algorithms are tools to reveal natural structures and identify interesting patterns in large-scale data. The clustering step is essential in data preprocessing and aims at dimension reduction of high-dimensional gene expression data in the framework. As one of the most widely used fuzzy clustering algorithms for microarrays, the fuzzy C-means clustering algorithm is utilised in the preprocessing of gene expression data. The significant advantage of the fuzzy C-means algorithm is its capability to deal with overlapping sample points. This is particularly useful for the clustering of gene expression data because the clusters of gene expression data may be highly intersected or even embedded. The fuzzy C-means algorithm is a soft clustering algorithm, with which each sample point in the cluster is characterized by its membership function [21]. Membership values $u_{ki}\in [0,1]$ and cluster centroids ${\textbf {c}}_k$ can be obtained by solving the following constrained optimization:

1 $$\begin{aligned} {\left\{ \begin{array}{ll} \displaystyle \min L(K,m)=\sum _{k=1}^K\sum _{i=1}^N (u_{ki})^m d^2({\varvec{y}}_i,{\varvec{c}}_k)\\ \displaystyle \text{ s.t. } \quad \sum _{k=1}^Ku_{ki}=1, i=1,...,N, k=1,...,K\\ \quad \quad \quad d^2({\varvec{y}}_i,{\varvec{c}}_k)=({\varvec{y}}_i-{\varvec{c}}_k)^T ({\varvec{y}}_i-{\varvec{c}}_k), \end{array}\right. } \end{aligned}$$

where *K* is the number of clusters, *m* is the fuzziness parameter, *N* is the number of genes, ${\varvec{y}}_i=(y_{i1},...,y_{iT})^{'}$ is a *T*-dimensional vector representing the *i*th gene with its *T* observations, and $d^2({\varvec{y}}_i,{\varvec{c}}_k)$ is the square of the Euclidian distance between ${\varvec{y}}_i$ and ${\varvec{c}}_k$. The details of optimization can be found in [25].

The number of clusters *K* and the fuzzifier *m* need to be chosen for fuzzy C-means clustering. The fuzzifier *m* can be estimated using the relation proposed by [26]. The selection of an optimal *K* is usually challenging and subjective. A method to choose the optimal number of clusters is to perform clustering in a range of cluster numbers and assess their biological relevance. The method of Gap statistics estimates the number of clusters by comparing the total within-cluster variation of a clustering solution with that of a reference distribution of data with no inherent clustering structure [27]. We will consider both the Gap statistics results and the biological relevance of the clusters to make informed decisions regarding the appropriate number of clusters for our study.

### Smoothing of target genes

The target genes can be determined according to the goal of the research. In this study, our focus lies specifically on the circadian rhythm. Li et al. [23] have described that “N-acetyltransferase gene (Aanat) is a potential target gene for melatonin rhythm regulation in rat pineal gland”. Kim et al. [28] have mentioned that the circadian rhythm of pineal melatonin requires the nocturnal increment of serotonin N-acetyltransferase (arylalkylamine N-acetyltransferase [AANAT]) protein. Several other studies have provided evidence demonstrating the significant role of the *Aanat* gene in regulating the circadian rhythm [29–32]. More specifically, we will focus on inferring gene regulatory relationships that contribute genetic information to *Aanat*.

Smoothing aims to capture essential patterns in the data by creating an approximating function. Local regression models are utilized, which fit curves and surfaces to time-series data by smoothing [33, 34]. This method is a generalization of the moving average and polynomial regression. The polynomial degree can be zero, one, and two (corresponding to kernel smoothing, linear polynomials, and quadratic polynomials, respectively).

The local regression model in the *i*th gene time-series data is

2 $$\begin{aligned} y_{it}=f(t)+\epsilon _t, t=1,\ldots ,T, \end{aligned}$$

where *f* is the regression function, *T* is the total number of time points, $\epsilon _t$ denotes a random error. The observations of the *i*th gene can be denoted as a vector ${\varvec{y}}_i=(y_{i1},\ldots ,y_{iT})^{'}$. The objective of smoothing is to estimate function *f*. A common assumption is that the random error follows the Gaussian distribution with a mean of 0 and a constant variance. We define a weight $\omega _t(x)=W(\Delta _t(x);\Delta _{(\alpha T)}(x))$, where $\Delta _t(x)=|x-x_t|$ and *W* is a tricube function:

$$\begin{aligned} W(x;u) = \left\{ \begin{array}{ll} (1-(x/u)^3)^3 &{} \text { for }0 \le x <u\\ 0 &{} \text { if }x \ge u \end{array} \right. \end{aligned}$$

For $\alpha \in (0,1]$, $\alpha T$ can be truncated to an integer and $\omega _t(x)=W(\Delta _t(x);\Delta _{[\alpha T]}(x))$. For $\alpha >1$, the weight is defined as $\omega _t(x)=W(\Delta _t(x);\Delta _{(T)}(x)\alpha )$. The estimated function’s smoothness depends on the neighbourhood parameter’s specification $\alpha$ [35]. The estimated function $\hat{f}$ becomes smoother when $\alpha$ increases. A generalized cross-validation is a feasible approach to deciding an optimal estimate of $\alpha$ [36, 37].

### Dynamic time warping distance

The distances between all pairs of genes and the smooth curve of the target genes are calculated to measure the similarity between all genes and the target genes. Dynamic time warping distance (DTW) is a standard algorithm for measuring similarity in time-series analysis. It can be used as a distance measure of expression values between gene pairs in microarray time-series data [38]. Suppose ${\varvec{Y}}$ and ${\varvec{Z}}$ are two time-series vectors of lengths $t_Y$ and $t_Z$, respectively. The traditional distance can be calculated if two time series have the same length, i.e., $t_Y=t_Z$:

3 $$\begin{aligned} d_{L_n}({\varvec{Y}},{\varvec{Z}})=\left( \sum _{j=1}^{t_Y} (Y_j-Z_j)^n \right) ^{\frac{1}{n}}, \end{aligned}$$

where *n* is a positive integer. These are called lock-step measures, which compute the distances of samples that are precisely at the exact temporal location. The Euclidean distance is Eq. (3) with $n=2$. But DTW can be used to deal with the scenario where the lengths of two time-series vectors are unequal. The principal idea of DTW is to find the path through the grid $p_1,\ldots ,p_s,\ldots ,p_h$ to minimize the total distance between ${\varvec{Y}}$ and ${\varvec{Z}}$, where $p_s=(u_s, v_s)$ and $u_s$ and $v_s$ are the values of the *s*th time point in ${\varvec{Y}}$ and ${\varvec{Z}}$, respectively (the figure illustration of DTW is presented in Additional file 1: Fig. S1). The best path between ${\varvec{Y}}$ and ${\varvec{Z}}$ is

4 $$\begin{aligned} \text{ min }(D({\varvec{Y}},{\varvec{Z}}))=\text{ min }\left( \frac{\sum _{s=1}^h d(p_s)w_s}{\sum _{s=1}^hw_s} \right) , \end{aligned}$$

where $d(p_s)$ is the distance between $u_s$ and $v_s$, $w_s$ is the weighting. There are different definitions of this weighting, such as symmetric weighting $w_s=(u_s-u_{s-1})+(v_s-v_{s-1})$ and asymmetric weighting $w_s=(u_s-u_{s-1})$ or $w_s=(v_s-v_{s-1})$, $s=(1,\ldots ,h)$. More details about the optimization can be found in [39].

Our rat gene expression data exhibits the same observed time points for each gene, enabling us to calculate both DTW and Euclidean distances. Additionally, we compute 20 other distances, including familiar metrics like Manhattan and Minkowski distances, along with specialized distances tailored for time series data, such as autocorrelation-based dissimilarity (ACF) and partial autocorrelation-based dissimilarity (PACF). These diverse distances are computed to facilitate comprehensive comparisons [40].

### Wilcoxon signed-rank test

Wilcoxon signed-rank test is a non-parametric hypothesis test to compare the locations of two populations using a set of matched samples. Using the Wilcoxon signed-rank test, Khan [41] has built an integrated tool, ArraySolver, for colour-coded graphical display and comparison of gene expression data. The null hypothesis of the Wilcoxon signed-rank test is that the differences between two groups of data have a mean of zero. We chose a p-value of 0.05 for statistical significance. If the p-value of the Wilcoxon signed-rank test is less than 0.05, the null hypothesis is rejected and the two groups are different.

We can obtain the number of significant genes in the selected cluster according to the results of the Wilcoxon signed-rank test. The Wilcoxon signed-rank test in this study is conducted to test whether the distance-based ranking of these genes is statistically the same as the ordinal ranking. In other words, one group of data is the distance-based ranking of genes in the selected cluster, and the other group of data is the ordinal ranking of genes. The optimal number of significant genes can be determined according to the p-values of the Wilcoxon signed-rank test. With the increase in the number of genes from ten, the number of significant genes is determined until the Wilcoxon test results are significant. As a result, we can identify the different number of significant genes using the Wilcoxon signed-rank test based on different types of distances.

### Dynamic vector auto-regressive models

This section introduces dynamic vector auto-regressive models for gene network construction. A VAR (vector auto-regressive) process of order one can be represented by:

$$\begin{aligned} {{\textbf {y}}}(t+1)={{\textbf {Ay}}}(t)+{{\textbf {B}}}+{\varvec{\epsilon }}(t), {\varvec{\epsilon }}(t) \sim N(0, \Sigma ), \end{aligned}$$

where ${{\textbf {y}}}(t)=(y_1(t),\ldots ,y_p(t))$, $t=1,\ldots ,T$ and ${{\textbf {A}}}=(a_{ij}),i,j=1,\ldots ,p$. If $a_{ij}\ne 0$, then the network includes an arc from ${{\textbf {y}}}_{j}(t)$ to ${{\textbf {y}}}_i(t+1)$, $i,j=1,\ldots ,p$.

We can obtain the ordinary least square estimates

$$\begin{aligned} \hat{{\textbf {A}}}_{OLS}=({\textbf {Y}}_{past}^T{\textbf {Y}}_{past})^{-1}{} {\textbf {Y}}_{past}^T{\textbf {Y}}_{future}, \end{aligned}$$

where $${\textbf {Y}}_{past}=\begin{bmatrix}{{\textbf {y}}}(1),\dots , {{\textbf {y}}}(T-1) \end{bmatrix}^{T}$$ and $${\textbf {Y}}_{future}=\begin{bmatrix}{{\textbf {y}}}(2),\dots ,{{\textbf {y}}}(T) \end{bmatrix}^{T}.$$ ${\textbf {Y}}_{past}^T{\textbf {Y}}_{past}$ is a $p \times p$ matrix with rank *T*. In the gene expression data, the number of genes *p* is usually much larger than the total time observations *T*, implying that the estimation matrix is sparse.

The regression coefficients reflect the influences between genes and they can be used to infer gene regulatory relationships. Statistical tests are required to obtain significant regression coefficients, which are included as edges in the gene regulatory relationships. Opgen-Rhein and Strimmer [10] proposed to test the corresponding partial correlation coefficients instead of the regression coefficients.

If ${{\textbf {y}}}_k(t)$ and ${{\textbf {y}}}_1(t+1)$ are reversed, we have

$$\begin{aligned}&{{\textbf {y}}}_1(t+1)=a_{1k}{{\textbf {y}}}_k(t)+\sum _{j=1,j \ne k}^p a_{1j}{{\textbf {y}}}_j(t)+b_1+\epsilon _1(t) \\&{{\textbf {y}}}_k(t)=a_{1k}^*{{\textbf {y}}}_1(t+1)+\sum _{j=1,j \ne k}^p a_{1j}^*{{\textbf {y}}}_j(t)+b_1^*+\epsilon ^*_1(t). \end{aligned}$$

Then, the partial correlation between the two variables is $\sqrt{a_{1k}a_{1k}^*}\text{ sgn }(a_{1k}^*)$. Specifically, this means that the regression coefficients are the partial correlations times the square root of the ratio of the partial variances.

The steps of constructing regulatory relationships are as follows:

1. Calculate VAR coefficients.
2. Convert the coefficients to partial correlations and then test the associated partial correlations.
3. Visualize the resulting network structure.

Opgen-Rhein and Strimmer [42] suggested using dynamical pairwise correlation to take account of the functional nature of the observed data. The dynamic correlation can be defined as:

5 $$\begin{aligned} \hat{\varrho }_{kl}= <f_{k}^S(t),f_{l}^S(t)>, \end{aligned}$$

where $f^S(t)=\frac{f(t)-<f(t),1>}{\sqrt{\text{ Var }(f(t))}}$ is the standardized functions of *f*(*t*). The functional inner product between two time series is defined as:

6 $$\begin{aligned} <g(t),h(t)>=\sum _{j=1}^T g(t_j)h(t_j) \frac{t_{j+1}-t_{j-1}}{2T}. \end{aligned}$$

The statistics test of dynamic partial correlation is the same as that of partial correlation. We can obtain the top significant edges and visualize the network.

## Results

### Data preprocessing

**Fig. 2:**
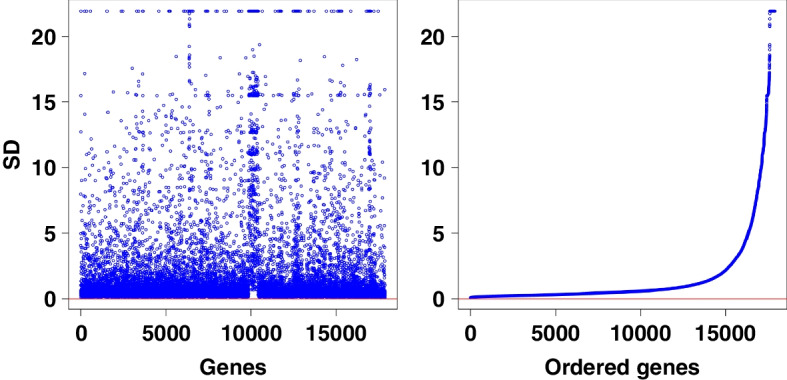
The normalized standard deviation of 17,879 genes

After removing 14,213 non-coding RNAs, 18,670 genes each with 480 observations were retained in the RNA extraction process. There are 791 genes whose standard deviation is zero. It is reasonable to remove these genes in the following analysis. The details of the distribution of the standard deviation of genes are presented in Fig. 2. It is seen from Fig. 2 that many genes have low standard deviations.

### The results of fuzzy C-means clustering

**Fig. 3:**
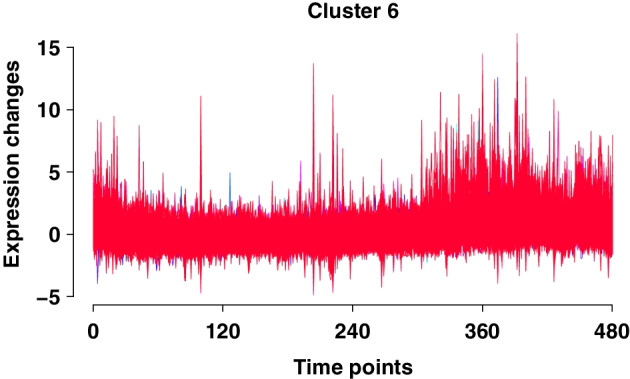
Cluster 6 of fuzzy C-means clustering results. The numbers on the x-axis (time, 0-480) correspond to the 480 time points during 24 h and y-axis represents the normalized expression value from the Mfuzz result

**Fig. 4:**
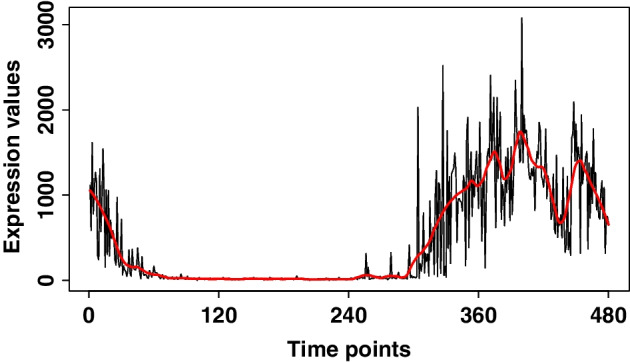
The optimal smooth for the Aanat gene

The fuzzy C-means clustering algorithm is applied to the gene expression data of 17,879 genes. We investigated the Gap statistics with the number of clusters ranging from 1 to 50. The estimated number of clusters is 47, which means that it has the maximum Gap statistic. However, the Gap statistic remains stable and increases slightly after the number of clusters is 9 (see Additional file 1: Fig. S2). The Gap statistic is not always reliable because it is sensitive to data structure and clustering algorithms [43]. Therefore, it is reasonable for us to choose the nine clusters. We present a total of nine clusters generated by Mfuzz [44] in Additional file 1: Fig. S3. In Fig. 3, we display a specific cluster that exhibits a stable trend during the middle of the time period and a subsequent increase in the last one-third of the total time period. This trend matches the pattern of gene *Aanat* (Fig. 4). Moreover, it is confirmed that gene *Aanat* is included in this cluster. The traditional way of analyzing gene expression data would focus on this specific cluster after obtaining the clustering results [21]. However, if we are interested in the regulatory relationships of a specific gene, such as the circadian gene (*Aanat*), focusing on one selected cluster is not enough because some relevant genes may be omitted due to their placement to other clusters. This motivates our new framework to select relevant genes not only from one specific cluster.

### Smoothing of the *Aanat* gene

In this study, our focus lies specifically on the circadian rhythm, and we have selected the *Aanat* gene as the target gene of interest. Figure 4 presents the trend of *Aanat* gene, which has a sharp decrease then remains stable and varies at the end of time periods. We fit a smooth curve for *Aanat* gene before measuring the similarity of the other genes and the *Aanat* gene. Since discrete observation time points are available for each gene, applying smoothing techniques provides the advantage of effectively extracting similar trends. We illustrated various smooth curves of the Aanat gene using quadratic polynomials in Additional file 1: Fig. S4. The optimal estimate of the neighborhood parameter $\alpha$ was determined to be 0.08 using the criterion of generalized cross-validation [36]. In Fig. 4, the red line represents the optimal smooth curve with the neighborhood parameter set to 0.08.

### Various distances between the smoothed *Aanat* gene and the total genes

**Fig. 5:**
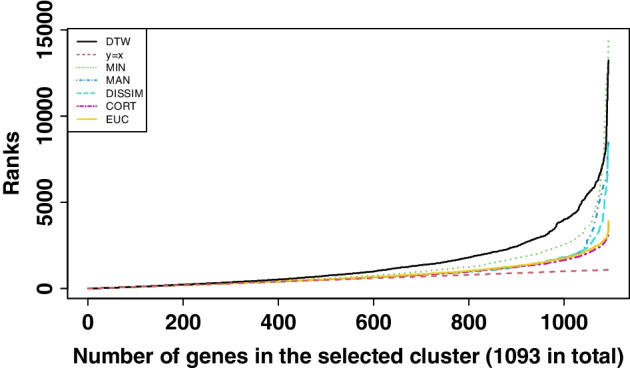
The corresponding ranks of genes in the selected cluster based on different distances. The red line is the straight line $y=x$ (reference line), which means the corresponding ranks of these 1,093 genes are 1 to 1,093

The distances between the 17,879 genes and the smooth curve of *Aanat* gene are calculated. Figure 5 presents the ranks of genes in the selected cluster (total 1,093 genes) for different distance metrics. For example, the rank of *Aanat* gene is seven according to the DTW distance (see Table 1). It is also noticed that the last rank of genes in the selected cluster is 13,225, far larger than the total number of genes in the selected cluster (1,093 genes). Therefore, we propose to use the Wilcoxon signed-rank test to test the differences between these 1093 genes in two different ranks to determine the number of significant genes. If the p-value is greater than 0.05, retain the null hypothesis, that is, assuming that the two groups of a certain number of genes are the same. With the increase in the number of genes from ten, the number of significant genes is determined until the Wilcoxon test results are significant. For example, for DTW distance, the Wilcoxon test is significant when the number of genes is within 253, implying that these 253 genes are statistically the same in the two types of ranking.

**Table 1:** The results of different distances with the row for DTW highlighted in bold being the best choice (the missed number of genes from other clusters are potentially significant genes)

Distance	Rank in	In Cluster 6		#missed
	total	Rank	#significant	genes from
	genes		genes	other clusters
1. Manhattan	1	1	823	187
2. Dissim	1	1	780	173
3. Cort	1	1	791	177
4. Euclidean	2	2	739	108
5. Cor	2	2	739	168
6. Fourier	2	2	741	171
7. Ccor	5	5	63	18
8. **DTW**	**7**	**7**	**253**	**51**
9. Minkowski	7	7	584	133
10. ACF	30	21	35	15
11. TAM	32	31	264	40
12. STS	34	24	34	16
13. SPEC	63	39	3	17
14. Periodogram	64	31	9	3
15. Infinite norm	97	96	313	174
16. INTPER	121	72	3	6
17. Frechet	166	156	278	135
18. NCD	4219	391	3	16
19. CDM	4952	396	3	65
20. PACF	17049	955	3	123

However, some genes would be omitted if we focus on only one selected cluster. Therefore, after obtaining the 253 genes according to DTW distance, check the distance values of the genes in other clusters to keep some omitted but relevant genes. We have found 51 such genes, whose distances to the smoothed *Aanat* gene are acceptable. Among these 51 genes, *Arntl* gene that belongs to cluster 5 is worth mentioning. According to [1], the *Arntl* gene is a core clock gene which controls aryl hydrocarbon receptor nuclear translocator-like (brain and muscle Arnt-like protein-1). They also mentioned other core clock genes, such as Per1, Per2 and Cry2 are all belong to the final selected 253 genes. Therefore, there are 304 genes in total are kept for further network construction.

Table 1 shows the ranking results of 20 distance metrics including the usual Manhattan, Euclidean, and Minkowski distances for time-series data. More details of the calculation of various distance metrics can be found in [40]. The rank in total genes in Table 1 means the rank of the *Aanat* gene in total genes. The rank value should not be too large because the distances are calculated based on the smoothed *Aanat* gene. Hence, the outcomes obtained from distance metrics 10 to 20 are deemed inadequate. These metrics include ACF (autocorrelation-based dissimilarity), Time Alignment Measurement (TAM), Short Time Series (STS), SPEC (Dissimilarity based on the generalized likelihood ratio test), Periodogram, Infinite norm, INTPER (Integrated periodogram based dissimilarity), Frechet, Normalized compress on based distance (NCD), Compression-based dissimilarity measure (CDM), and Partial autocorrelation-based dissimilarity (PACF). The analysis reveals that the first six distance metrics, namely Manhattan, Dissim, Cort (Dissimilarity index combining temporal correlation and raw value behaviors), Euclidean, Cor (Dissimilarities based on Pearson’s correlation), and Fourier (Distance based on the Fourier Discrete Transform), still yield a considerable number of significant genes according to the rank test. This indicates ineffective dimension reduction. Consequently, only three distance metrics remain for further consideration: Ccor (cross-correlation), DTW, and Minkowski. Notably, compared to the DTW distance, Ccor demonstrates significantly fewer missed genes from other clusters, while Minkowski produces an excessive number of significant genes. Therefore, it can be concluded that, in this study, DTW is a superior distance measure compared to other distances.

### Regulatory relationships

**Fig. 6:**
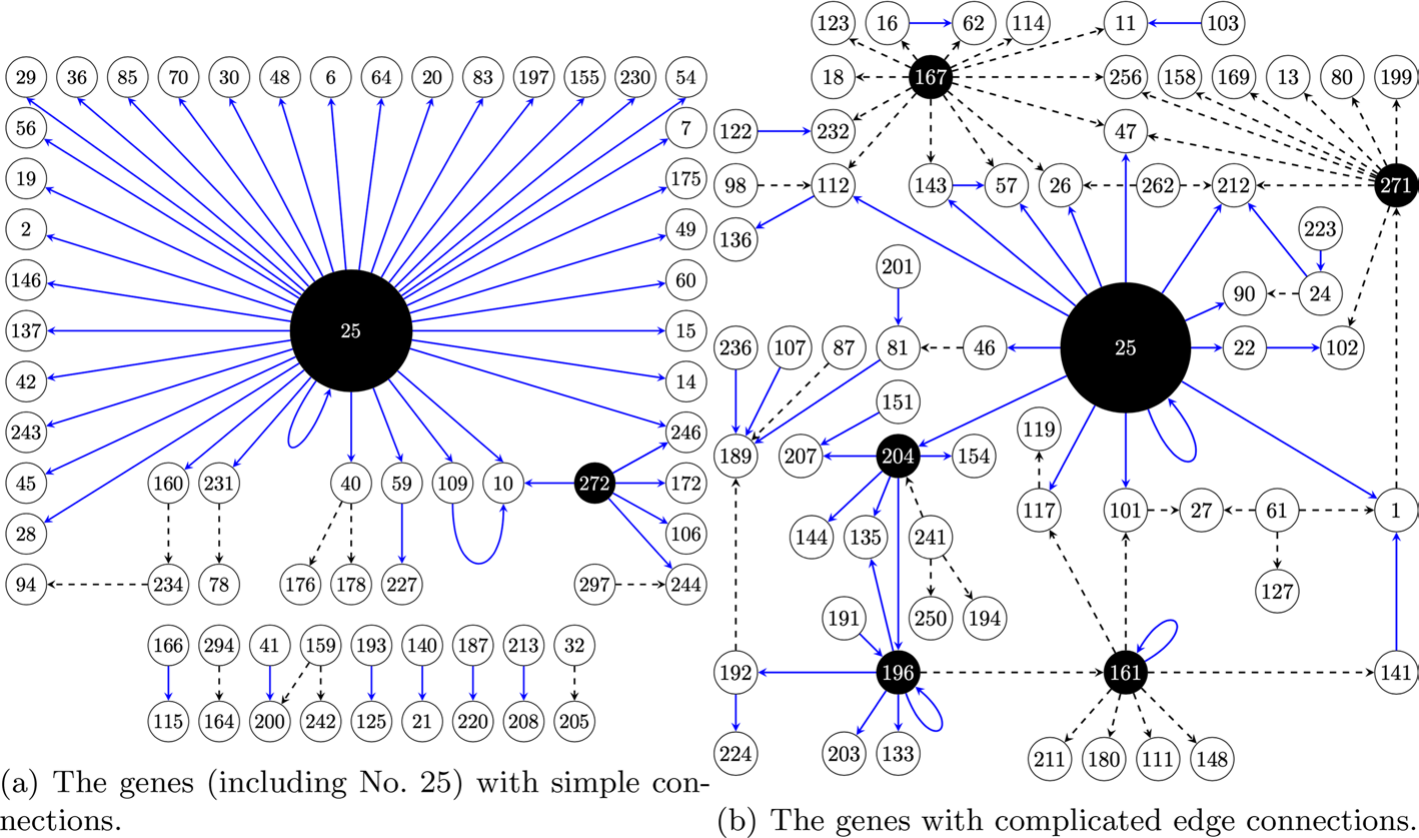
The relationships based on the dynamic VAR. 150 top significant edges are presented. The numbers corresponding to the ID of genes and the complete list can be found in Additional file 1: Table 1. The blue solid line represents a positive correlation, while the black dashed line indicates a negative correlation between two genes

We visualize the network from dynamic VAR based on the final selected genes in Fig. 6. The undepicted of these two parts can be seen in Additional file 1: Fig. S5. With the most number of edges, the *Pde10a* gene (number 25) deserves further research. Wolloscheck et al. [45] have shown that *Pde10a* is highly expressed in retinal neurons including photoreceptors. The levels of *Pde10a* transcript and protein display daily rhythms, which could be seen in preparations of the whole retina. Other studies have revealed the role of *Pde10a* gene in the circadian regulation [46, 47]. The network graph constructed from the dynamic partial correlation is presented in the Additional file 1: Fig. S6.

**Fig. 7:**
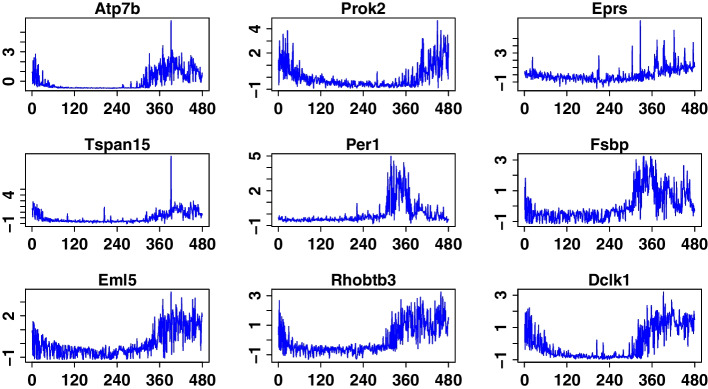
The plot of related genes with the Aanat gene. The x-axis is the time points, and the y-axis is the expression values

We selected the top ten genes that control or relate to the *Aanat* gene. Figure 7 presents the trends of these ten genes over a period of 24 h. Genes *Pde10a, Tspan15, Per1* and *Fsbp* control the *Aanat* gene positively. Also, genes *Atp7b, Prok2, Eprs, Eml5, Rhobtb3* and *Dclk1* affect the *Aanat* gene negatively. For the gene *Atp7b*, it has been known to have a direct effect on a circadian rhythm [48, 49]. Night-specific ATPase (PINA) is generated by an intronic promoter in *Atp7b* gene. *Prok2* gene encodes a protein expressed in the suprachiasmatic nucleus (SCN) circadian clock, which may function as the output component of the circadian clock. Prosser et al. [50] have claimed that *Prok2* gene is essential for regulating circadian behaviour by the suprachiasmatic nuclei. Martin et al. [51] have also shown that the prokineticin 2 (*Prok2*), and Prok2 receptor (*Prokr2*) have emerged as critical regulators of reproduction in both mice and humans. Vriend et al. [52] have indicated that *Rhobtb3* plays an important role in light/dark-induced adrenergic modulation of pineal function. Brüning et al. [53] have indicated that threonine-protein kinase DCLK1 peaks in activity at the wake-sleep transition. Overall, genes *Pde10a, Atp7b, Prok2, Per1, Rhobtb3*, and *Dclk1* have been revealed to be essential for the regulation of circadian behaviour

No direct evidence has been observed that the genes *Eprs, Eml5, Tspan15* and *Fsbp* are related to the circadian rhythm. For the *Eprs* gene, Yao et al. [54] have mentioned that it has a unique role in GAIT-mediated translational control as it is solely responsible for recognition and interaction with GAIT elements in target mRNAs. *Eml5* plays a role in regulating cytoskeletal rearrangements during neuronal development and in adult brain [55]. While there are research efforts about the gene *Tspan15*, more research needs to be done on the relationship between the *Tspan15* gene and a circadian rhythm [56, 57]. Moreover, gene *Fsbp* (Fibrinogen Silencer Binding Protein) is related to the Alzheimer’s disease [58].

### Validation on public datasets

To demonstrate the applicability of our proposed method, we conducted validation using publicly available datasets known as BEELINE datasets [59]. Specifically, we utilized the experimental single-cell RNA-Seq datasets of hHep (Human Mature Hepatocytes) [60]. The dataset comprised 425 single-cell transcriptomes collected at various time points during the differentiation process. These time points corresponded to the following cell types: induced pluripotent stem (iPS) cells, definitive endoderm (DE), hepatic endoderm (HE), immature hepatoblast-like (IH) cells, and mature hepatocyte-like (MH) cells. The time points were day 0, day 6, day 8, day 14, and day 21, respectively. To align with our method, which relies on time-series datasets for inferring gene regulatory networks, we constructed pseudo-time-series gene expression datasets based on time-lapse information. From this process, we retained 11,515 genes with 425 observations/cells for our comprehensive analysis. According to our framework (Fig. 1), we did the clustering first and then selected the specific cluster with the target gene. In this particular instance, we selected *CER1* as the target gene due to previous investigations of its regulatory relationships by Camp et al. [60], enabling meaningful comparisons. The specific details of each step were omitted. However, the final regulatory relationships for the *CER1* gene have been established. The top three genes associated with *CER1* are *HAS2*, *CCL2*, and *EOMES*. These relationships were validated in Extended Data Figure 2 by Camp et al. [60].

## Discussion

Gene expression datasets are usually large and complex and thus require effective methods to process and analyze. The hybrid framework presented in this paper is a generic method in the sense that it is not limited to a specific type of dataset. Thus, in principle, it is applicable in inferring other gene regulatory relationships. In this study, circadian genes are our interests and the *Aanat* gene is our target gene. More details about selecting the *Aanat* as the target gene have been described in the Sect. Smoothing of target genes. While only one target gene is considered in this study, the hybrid framework can be extended for more than one target gene. The smoothing process for two or more target genes would be the same as for a single target gene. DTW distance is also recommended for measuring gene similarity because of its advantage in comparing the similarity between two time-series data. What does need to be considered in dealing with more than one target gene is how to decide the number of selected genes according to the distances between two or more target genes and all genes. Conceptually, the number of significant genes can still be determined based on the Wilcoxon signed-rank test. This deserves further research.

For the choice of the number of clusters, we have added the results from Gap statistics. But we understand that the result of Gap statistics is not always reliable, and it just gives us a reference to make decisions. As a novel aspect of our hybrid framework, we chose to measure the similarity between all genes and the smoothness of the target gene, rather than the similarity between all genes and the target gene itself. The advantage of smoothing is that it can capture the pattern of the target gene, and then reflect the actual trend to obtain the most relevant genes. As a practical and traditional approach, local regression models are adopted for the smoothing of time-series data. Further investigations are required to evaluate the performance of other smoothing methods (such as bin smoothing, kernel smoothing, and local weighted regression) in this hybrid framework for inferring gene regulatory relationships.

## Conclusions

A new hybrid framework has been proposed for inferring specific gene regulatory relationships. Circadian or clock genes are the targets for this study and the approach of this framework can be migrated to the analysis of any other circadian genes. Our hybrid framework combines the fuzzy C-means method and dynamic time warping distance method. It can deal with the high-dimensional problem, which always exists in the gene expression data. Dynamic VAR has been proposed to construct the circadian gene regulatory relationships based on the experimental gene expression data of rats. Genes *Pde10a, Atp7b, Prok2, Per1, Rhobtb3* and *Dclk1* are standout for the regulation of circadian behaviour. Moreover, this framework is generic and can be used for other gene expression datasets.

### Supplementary Information

**Additional file 1.** Supplementary figures, Supplementary tables.

## Data Availability

The raw gene expression data are available for download at Sequence Read Archive (SRA) (SRR18934928 - SRR18935407) on the National Library of Medicine website. More details about this dataset can be found in [23]. The R code is freely available at https://github.com/hu038/Circadian_gene_relationships.

## Abbreviations

DTW: Dynamic time warping
FDR: False discovery rate
DBNs: Dynamic Bayesian networks
LASSO: Least absolute shrinkage and selection operator
GGMs: Graphical Gaussian models
PCA: Principal-component analysis
NETS: Network estimation for time series
VAR: Vector auto-regressive

## References

[1] Li JZ, Bunney BG, Meng F, Hagenauer MH, Walsh DM, Vawter MP, Evans SJ, Choudary PV, Cartagena P, Barchas JD (2013). Circadian patterns of gene expression in the human brain and disruption in major depressive disorder. Proc Natl Acad Sci.

[2] Zhang R, Lahens NF, Ballance HI, Hughes ME, Hogenesch JB (2014). A circadian gene expression atlas in mammals: implications for biology and medicine. Proc Natl Acad Sci.

[3] Caba M, González-Mariscal G, Meza E (2018). Circadian rhythms and clock genes in reproduction: insights from behavior and the female rabbit’s brain. Front Endocrinol.

[4] Steuer R (2006). On the analysis and interpretation of correlations in metabolomic data. Brief Bioinform.

[5] Benjamini Y, Hochberg Y (1995). Controlling the false discovery rate: a practical and powerful approach to multiple testing. J Statist Soc B.

[6] Benjamini Y (2010). Discovering the false discovery rate. J R Stat Soc Series B (Stat Methodol).

[7] Strimmer K (2008). A unified approach to false discovery rate estimation. BMC Bioinform.

[8] Opgen-Rhein R, Strimmer K (2007). From correlation to causation networks: a simple approximate learning algorithm and its application to high-dimensional plant gene expression data. BMC Syst Biol.

[9] Martin S, Zhang Z, Martino A, Faulon J-L (2007). Boolean dynamics of genetic regulatory networks inferred from microarray time series data. Bioinformatics.

[10] Opgen-Rhein R, Strimmer K (2007). Learning causal networks from systems biology time course data: an effective model selection procedure for the vector autoregressive process. BMC Bioinform.

[11] Nagarajan R, Scutari M, Lèbre S. Bayesian networks in r Springer. 2013;122:125–7.

[12] Qiu J, Wang H, Hu L, Yang C, Zhang T (2021). Spatial transmission network construction of influenza-like illness using dynamic bayesian network and vector-autoregressive moving average model. BMC Infect Dis.

[13] Peng J, Wang P, Zhou N, Zhu J (2009). Partial correlation estimation by joint sparse regression models. J Am Stat Assoc.

[14] Friedman J, Hastie T, Tibshirani R (2008). Sparse inverse covariance estimation with the graphical lasso. Biostatistics.

[15] Fu WJ (1998). Penalized regressions: the bridge versus the lasso. J Comput Graph Stat.

[16] Krämer N, Schäfer J, Boulesteix A-L (2009). Regularized estimation of large-scale gene association networks using graphical gaussian models. BMC Bioinform.

[17] D’Angelo GM, Rao DC, Gu CC. Combining least absolute shrinkage and selection operator (lasso) and principal-components analysis for detection of gene-gene interactions in genome-wide association studies. In: BMC Proceedings, vol. 3, pp. 1–5 (2009). BioMed Central 10.1186/1753-6561-3-s7-s62 PMC2795963 20018056

[18] Barigozzi M, Brownlees C (2019). Nets: network estimation for time series. J Appl Economet.

[19] Ajmal HB, Madden MG (2020). Inferring dynamic gene regulatory networks with low-order conditional independencies-an evaluation of the method. Stat Appl Genet Mol Biol.

[20] Lèbre S (2009). Inferring dynamic genetic networks with low order independencies. Stat Appl Genet Mol Biol.

[21] Oyelade J, Isewon I, Oladipupo F, Aromolaran O, Uwoghiren E, Ameh F, Achas M, Adebiyi E (2016). Clustering algorithms: their application to gene expression data. Bioinform Biol Insights.

[22] Kerr G, Ruskin HJ, Crane M, Doolan P (2008). Techniques for clustering gene expression data. Comput Biol Med.

[23] Li T, Liu Z, Wang Y, Zuo D, Wang S, Ju H, Wang S, Yanping X, Ling Y, Liu C (2022). Multiplexed imaging method to explore complete targeting regulatory relationships among circadian genes for insomnia treatment. Front Neurosci.

[24] Liu Z, Gao J, Li T, Jing Y, Xu C, Zhu Z, Zuo D, Chen J (2022). A novel approach grntste to reconstruct gene regulatory interactions applied to a case study for rat pineal rhythm gene. Sci Rep.

[25] Dembele D, Kastner P (2003). Fuzzy c-means method for clustering microarray data. Bioinformatics.

[26] Schwämmle V, Jensen ON (2010). A simple and fast method to determine the parameters for fuzzy c-means cluster analysis. Bioinformatics.

[27] Tibshirani R, Walther G, Hastie T (2001). Estimating the number of clusters in a data set via the gap statistic. J R Stat Soc Series B (Stat Methodol).

[28] Kim T-D, Woo K-C, Cho S, Ha D-C, Jang SK, Kim K-T (2007). Rhythmic control of aanat translation by hnrnp q in circadian melatonin production. Genes Dev.

[29] Foulkes NS, Whitmore D, Sassone-Corsi P (1997). Rhythmic transcription: the molecular basis of circadian melatonin synthesis. Biol Cell.

[30] Simonneaux V, Sinitskaya N, Salingre A, Garidou ML, Pévet P (2006). Rat and syrian hamster: two models for the regulation of aanat gene expression. Chronobiol Int.

[31] Ciarleglio CM, Ryckman KK, Servick SV, Hida A, Robbins S, Wells N, Hicks J, Larson SA, Wiedermann JP, Carver K (2008). Genetic differences in human circadian clock genes among worldwide populations. J Biol Rhythms.

[32] Tosini G, Pozdeyev N, Sakamoto K, Iuvone PM (2008). The circadian clock system in the mammalian retina. BioEssays.

[33] Cleveland WS, Loader C. Smoothing by local regression: principles and methods. In: Statistical theory and computational aspects of smoothing: proceedings of the COMPSTAT’94 satellite meeting held in Semmering, Austria, 27–28 August 1994, Springer; 1996. pp. 10–49.

[34] Loader C. Smoothing: local regression techniques. Springer; 2012. p. 571–96.

[35] Cleveland W, Grosse E, Shyu W. Local regression models. chapter 8 in statistical models in s (jm chambers and tj hastie eds.), Wadsworth & Brooks/Cole, Pacific Grove, CA; 1992. p. 608

[36] Golub GH, Heath M, Wahba G (1979). Generalized cross-validation as a method for choosing a good ridge parameter. Technometrics.

[37] Wang X. fANCOVA: nonparametric analysis of covariance. (2020). R package version 0.6-1. https://CRAN.R-project.org/package=fANCOVA

[38] Yang AC, Hsu H-H, Lu M-D, Tseng VS, Shih TK (2014). Prediction of regulatory gene pairs using dynamic time warping and gene ontology. Int J Data Min Bioinform.

[39] Giorgino T (2009). Computing and visualizing dynamic time warping alignments in r: the dtw package. J Stat Softw.

[40] Mori U, Mendiburu A, Lozano JA (2016). Distance measures for time series in r: the TSdist package. R J.

[41] Khan HA (2004). Arraysolver: an algorithm for colour-coded graphical display and wilcoxon signed-rank statistics for comparing microarray gene expression data. Comp Funct Genomics.

[42] Opgen-Rhein R, Strimmer K. Using regularized dynamic correlation to infer gene dependency networks from time-series microarray data. In: Proceedings of the 4th international workshop on computational systems biology (WCSB 2006), Tampere, vol. 4, pp. 73–76 2006;. Citeseer

[43] Mohajer M, Englmeier K-H, Schmid VJ. A comparison of gap statistic definitions with and without logarithm function, 2011. arXiv preprint arXiv:1103.4767

[44] Kumar L, Futschik ME (2007). Mfuzz: a software package for soft clustering of microarray data. Bioinformation.

[45] Wolloscheck T, Spiwoks-Becker I, Rickes O, Holthues H, Spessert R (2011). Phosphodiesterase10a: abundance and circadian regulation in the retina and photoreceptor of the rat. Brain Res.

[46] Spiwoks-Becker I, Wolloscheck T, Rickes O, Kelleher DK, Rohleder N, Weyer V, Spessert R (2011). Phosphodiesterase 10a in the rat pineal gland: localization, daily and seasonal regulation of expression and influence on signal transduction. Neuroendocrinology.

[47] Beker MC, KiliÇ E (2021). The role of circadian rhythm in the regulation of cellular protein profiles in the brain. Turk J Med Sci.

[48] Borjigin J, Sun X, Wang MM. The role of pina in copper transport, circadian rhythms, and wilson’s disease. In: Handbook of copper pharmacology and toxicology. Springer; 2002. p. 201–7.

[49] Ahmed S, Deng J, Borjigin J (2005). A new strain of rat for functional analysis of pina. Mol Brain Res.

[50] Prosser HM, Bradley A, Chesham JE, Ebling FJ, Hastings MH, Maywood ES (2007). Prokineticin receptor 2 (prokr2) is essential for the regulation of circadian behavior by the suprachiasmatic nuclei. Proc Natl Acad Sci.

[51] Martin C, Balasubramanian R, Dwyer AA, Au MG, Sidis Y, Kaiser UB, Seminara SB, Pitteloud N, Zhou Q-Y, Crowley WF (2011). The role of the prokineticin 2 pathway in human reproduction: evidence from the study of human and murine gene mutations. Endocr Rev.

[52] Vriend J, Liu W, Reiter RJ (2017). The pineal gland: a model for adrenergic modulation of ubiquitin ligases. PLoS ONE.

[53] Brüning F, Noya SB, Bange T, Koutsouli S, Rudolph JD, Tyagarajan SK, Cox J, Mann M, Brown SA, Robles MS (2019). Sleep-wake cycles drive daily dynamics of synaptic phosphorylation. Science.

[54] Yao P, Potdar AA, Arif A, Ray PS, Mukhopadhyay R, Willard B, Xu Y, Yan J, Saidel GM, Fox PL (2012). Coding region polyadenylation generates a truncated trna synthetase that counters translation repression. Cell.

[55] O’Connor V, Houtman S, De Zeeuw C, Bliss T, French P (2004). Eml5, a novel wd40 domain protein expressed in rat brain. Gene.

[56] Saftig P, Lichtenthaler SF (2015). The alpha secretase adam10: a metalloprotease with multiple functions in the brain. Prog Neurobiol.

[57] Bi Y, Cui D, Xiong X, Zhao Y (2021). The characteristics and roles of β-trcp1/2 in carcinogenesis. FEBS J.

[58] Lau K-F, Perkinton MS, Rodriguez L, McLoughlin DM, Miller CC (2010). An x11α/fsbp complex represses transcription of the gsk3β gene promoter. NeuroReport.

[59] Pratapa A, Jalihal AP, Law JN, Bharadwaj A, Murali TM (2020). Benchmarking algorithms for gene regulatory network inference from single-cell transcriptomic data. Nat Methods.

[60] Camp JG, Sekine K, Gerber T, Loeffler-Wirth H, Binder H, Gac M, Kanton S, Kageyama J, Damm G, Seehofer D (2017). Multilineage communication regulates human liver bud development from pluripotency. Nature.

