# Supplementary material for: Inferring circadian gene regulatory relationships from gene expression data with a hybrid framework
Source: BMC Bioinformatics. 2023 Sep 26;24:362. doi: 10.1186/s12859-023-05458-y (PMC10521455; doi:10.1186/s12859-023-05458-y)
Supplement: Supplementary file 1 — Additional file 1. Supplementary figures, Supplementary tables. [file 12859_2023_5458_MOESM1_ESM.pdf]

Supplementary Figures

Inferring Circadian Gene Regulatory Relationships from Gene Expression Data with a Hybrid Framework

Shuwen Hu[1,2#], Yi Jing[3#], Tao Li[4#], You-Gan Wang[5], Zhenyu Liu[6], Jing Gao[6*] and Yu-Chu Tian[1*]

[1] School of Computer Science, Queensland University of Technology, Brisbane QLD 4001, Australia
[2] Agriculture and Food, CSIRO, St Lucia, QLD 4067, Australia
[3] Faculty of Science, The University of New South Wales, Sydney NSW 2052, Australia
[4] School of Life Sciences, Inner Mongolia Agricultural University, Hohhot 010018, China
[5] Institute for Learning Sciences and Teacher Education, Australian Catholic University, Brisbane QLD 4000, Australia
[6] School of Computer and Information Engineering, Inner Mongolia Agricultural University, Hohhot 010018, China

[#]Co-First Author. [*]Corresponding author: gaojing@imau.edu.cn; y.tian@qut.edu.au

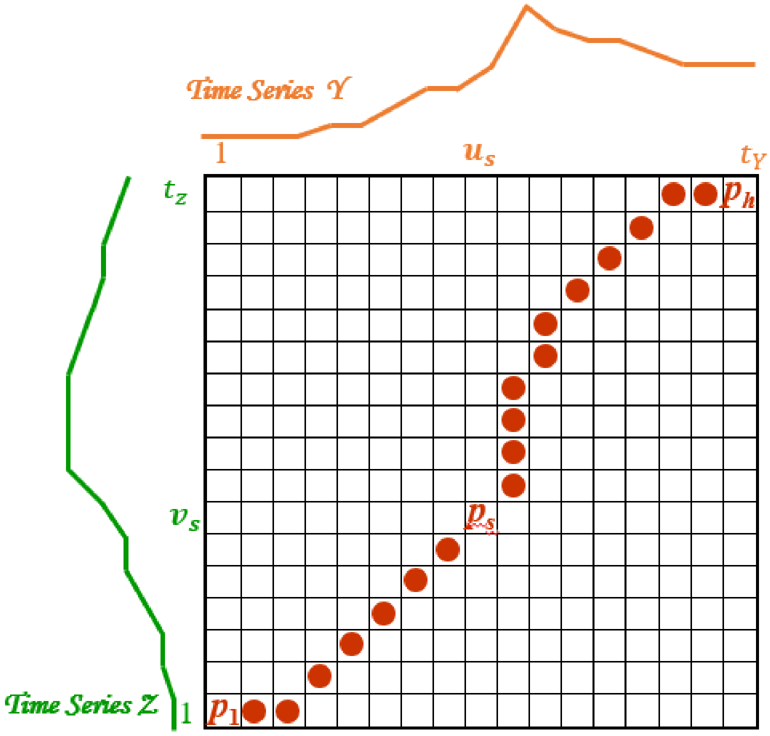

Figure S1: The illustration of the DTW algorithm.

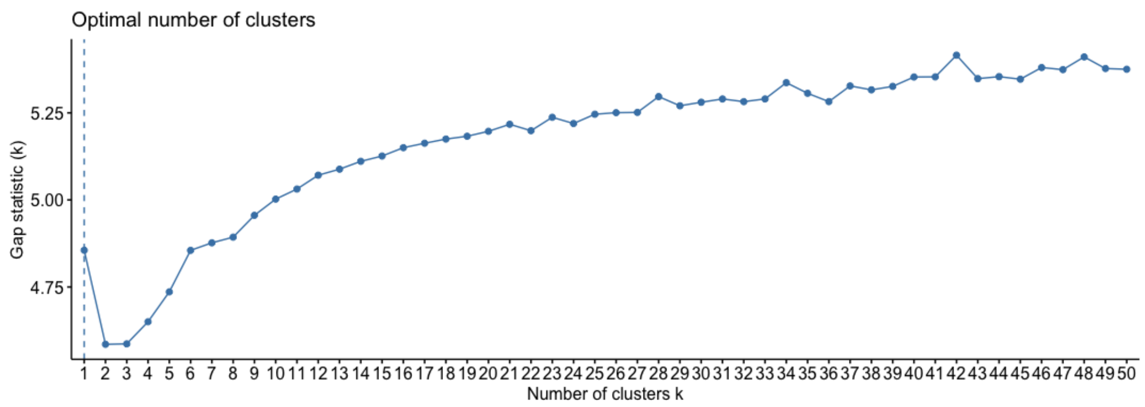

Figure S2: The results gap statistics.

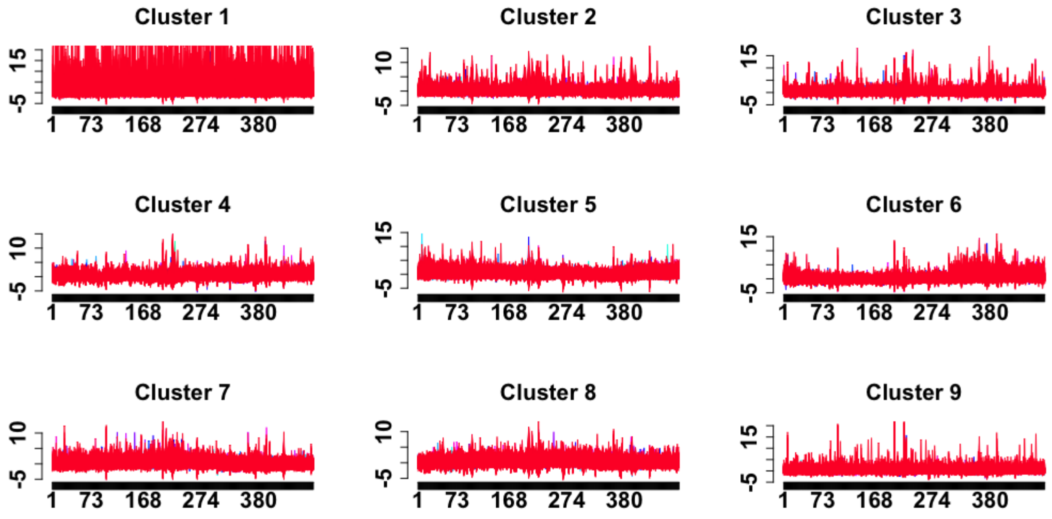

Figure S3: The results of fuzzy c-means clustering.

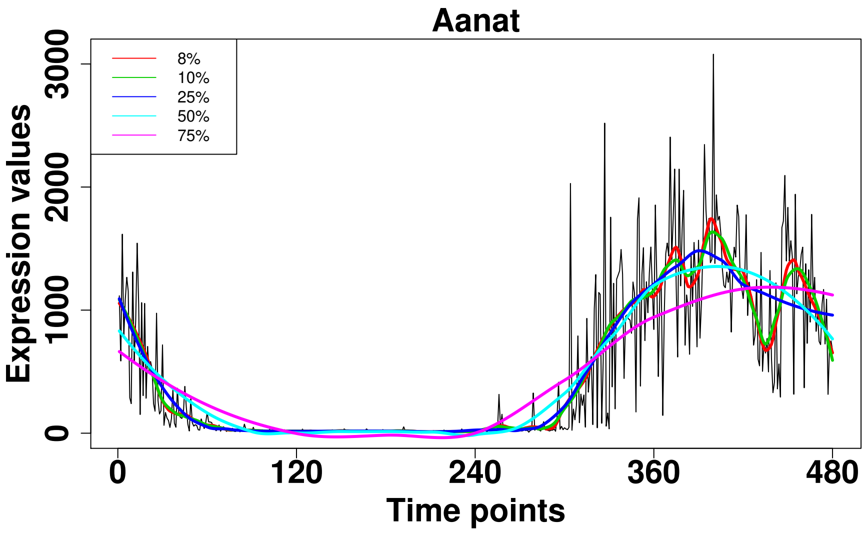

Figure S4: The different span of smooth for the Aanat gene.

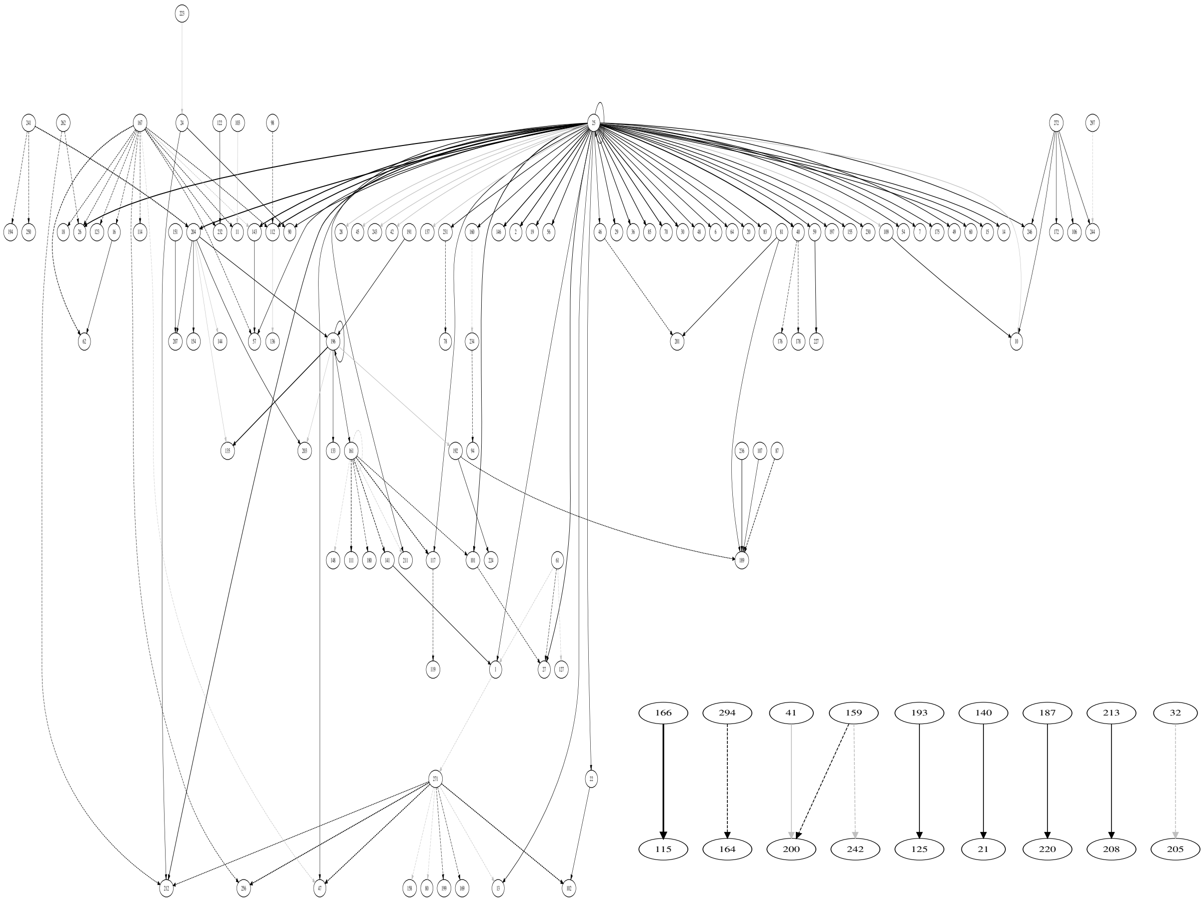

Figure S5: The relationships based on the dynamic VAR. 150 top significant edges are presented.

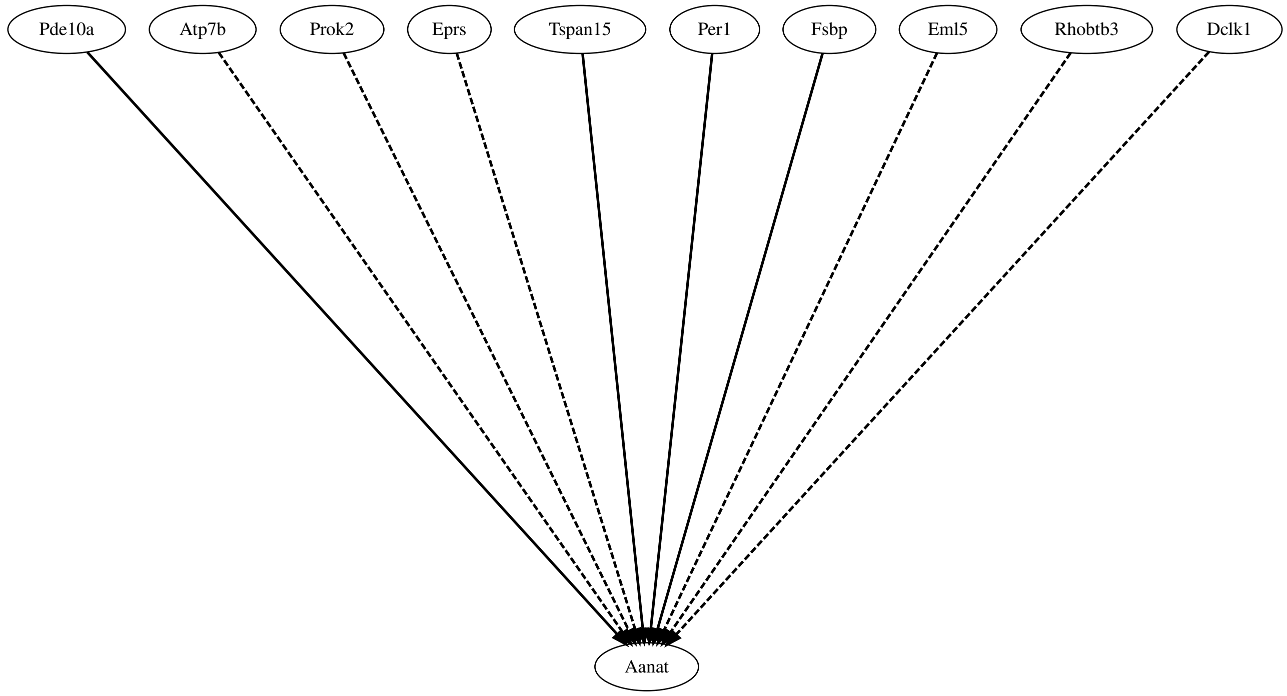

Figure S6: The network of related genes with the Aanat gene according to dynamic VAR.

Number	Gene	Number	Gene	Number	Gene
1	Fcer1a	2	Galnt16	6	Camk1g
7	Aanat	10	Tbc1d1	11	Reep2
13	Lamb3	14	Fam161a	15	Dnm2
16	Etnk1	18	Mcam	19	Kctd3
20	Tpcn1	21	Pomgnt1	22	Hhip
24	Tspan15	25	Pde10a	26	Xbp1
27	Fkbp5	28	Whrn	29	Irak2
30	Sik2	32	Tyro3	36	Grm1
40	Atp7b	41	Nphp4	42	Frmpd1
45	Cd24	46	Mbnl2	47	Rxfp2
48	Gxylt1	49	Igf1r	54	Klhl30
56	Nfatc1	57	Hspa5	59	Dclk3
60	Nacad	61	Rcan1	62	Rit1
64	Nptx1	70	Ptch1	78	Mt1
80	Mpp6	81	Xpot	83	Fam160b1
85	Man2a1	87	Wfikkn2	90	Chst2
94	Slc15a1	98	Trank1	101	Rhobtb3
102	Cdc5l	103	Arfgef3	106	Gnaz
107	Gls2	109	Bsx	111	Amd1
112	Abca1	114	Pvr	115	Abcf1
117	Zhx1	119	Tinagl1	122	Ell2
123	Itpkc	125	Padi4	127	Lcp1
133	Bend3	135	Spata2L	136	Wdr41
137	Mapk6	140	Tpo	141	Rab3ip
143	Rock2	144	Gdf7	146	Lamb1
148	Gls	151	Tjp2	154	Midn
155	Ankrd52	158	Irf8	159	Elfn2
160	Slc7a5	161	Kcnv2	164	Rgr
166	Wdpcp	167	Eml5	169	Draxin
172	Tatdn2	175	Cyth3	176	Soat2
178	Gstt3	180	Herc4	187	Noct
189	Hcrtr1	191	Usp16	192	Fhdc1
193	Il20ra	194	Ctps1	196	Ppp1r15a
197	Cep170b	199	Lrrc1	200	Tmem233
201	Tent5b	203	Amotl2	204	Rbm14
205	Hsph1	207	Cbx4	208	Hars2
211	Tbc1d30	212	Plcl2	213	Fzd4
220	Thap4	223	Hspa1b	224	Padi3
227	Ccrl2	230	Cacna1g	231	Galnt7
232	Ankmy2	234	Eprs	236	Pcdh1
241	Rgs2	242	Mxi1	243	Lrrc4
244	Tmem108	246	Foxp4	250	Cited4
256	Tuba1b	262	Inpp5a	271	Prok2
272	Timp4	294	Myrfl	297	Wasf1

Table S1: The corresponding gene for the numbers in network.
